# A Search for *Trypanosoma brucei rhodesiense* Diagnostic Antigens by Proteomic Screening and Targeted Cloning

**DOI:** 10.1371/journal.pone.0009630

**Published:** 2010-03-10

**Authors:** Theresa Manful, Julius Mulindwa, Fernanda M. Frank, Christine E. Clayton, Enock Matovu

**Affiliations:** 1 Zentrum für Molekulare Biologie der Universität Heidelberg, DKFZ-ZMBH Alliance, Heidelberg, Germany; 2 Faculty of Veterinary Medicine, Makerere University, Kampala, Uganda; 3 Cátedra de Inmunología IDEHU (UBA-CONICET), Facultad de Farmacia y Bioquímica (UBA), Buenos Aires, Argentina; New York University School of Medicine, United States of America

## Abstract

**Background:**

The only available diagnostic method for East African trypanosomiasis is light microscopy of blood samples. A simple immunodiagnostic would greatly aid trypanosomiasis control.

**Methodology and Principal Findings:**

To find trypanosome proteins that are specifically recognised by sera from human sleeping sickness patients, we have screened the *Trypanosoma brucei brucei* proteome by Western blotting. Using cytosolic, cytoskeletal and glycosomal fractions, we found that the vast majority of abundant trypanosome proteins is not specifically recognised by patient sera. We identified phosphoglycerate kinase (PGKC), heat shock protein (HSP70), and histones H2B and H3 as possible candidate diagnostic antigens. These proteins, plus paraflagellar rod protein 1, rhodesain (a cysteine protease), and an extracellular fragment of the *Trypanosoma brucei* nucleoside transporter *TbNT*10, were expressed in *E. coli* and tested for reactivity with patient and control sera. Only *Tb*HSP70 was preferentially recognized by patient sera, but the sensitivity and specificity were insufficient for use of *Tb*HSP70 alone as a diagnostic. Immunoprecipitation using a native protein extract revealed no specifically reacting proteins.

**Conclusions:**

No abundant *T. brucei* soluble, glycosomal or cytoskeletal protein is likely to be useful in diagnosis. To find useful diagnostic antigens it will therefore be necessary to use more sophisticated proteomic methods, or to test a very large panel of candidate proteins.

## Introduction

Human African trypanosomiasis is transmitted by Tsetse flies and occurs in a broad band from south of the Sahara to Zimbabwe. It occurs in two major forms, caused by *Trypanosoma brucei gambiense* (*T. b. gambiense*) in West and Central Africa and *Trypanosoma brucei rhodesiense* (*T. b. rhodesiense*) in East and Southern Africa. The initial symptoms are very non-specific, so the disease may not be diagnosed until the parasites have penetrated the brain, at which point treatment becomes both difficult and dangerous [1], [2]. A sustained control programme is required if new trypanosomiasis epidemics are to be avoided [3]. For primary screening, a low-cost, simple and rapid diagnosis method with high sensitivity is required: microscopic confirmation and a DNA-based subspecies determination could then be restricted to positive cases.

African trypanosomes are coated by Variant Surface Glycoprotein (VSG), and have many *VSG* genes and pseudogenes which are sequentially expressed during infection. Although there are enormous variations in repertoire between isolates, most *T. b. gambiense* isolates possess and express at least one of three particular *VSG* genes. This enabled the development, for *T. b. gambiense* infection, of the rapid Card Agglutination Test for Trypanosomiasis (CATT); this relies on the presence of antibodies to those VSGs in patients [4]. Since all available sleeping sickness treatments have severe side-effects, this test is always followed by microscopic examination for live parasites [4], [5]. Microscopy of thick films has low sensitivity (10,000 parasites per ml) [4] and scanning a slide can take 10 minutes; use of haematocrit tubes increases the sensitivity, but is more time consuming [4], so microscopy is not really practical as a large-scale screening method. The CATT test is also not 100% sensitive: false negatives arise because some *T. b. gambiense* do not express the relevant VSGs. Worse, no shared VSGs have been found for *T. b. rhodesiense*, so there is no field-adapted screening test for East African trypanosomiasis. DNA amplification methods [6] [7] are highly sensitive for detection of trypanosomes, and easily capable of distinguishing between species and subspecies. The most sensitive method to date, loop-mediated isothermal amplification (LAMP) [7], relies on amplification of a multicopy transposon-like sequence for detection of *T. brucei*, with more specific primers for subspecies determination. There is however no immediate prospect of the use of amplification methods for surveillance of trypanosomiasis in Africa. They are too expensive and time-consuming, and require too much training for implementing personnel.

The simplest and cheapest rapid tests for *T. b. rhodesiense* are likely – like the CATT - to rely on antibodies to trypanosome antigens in sera. The ideal candidate for development of a serodiagnostic tool should be an immunogenic protein that is consistently expressed by the bloodstream-form trypanosomes. All trypanosome proteins are potential candidates, since they are all released into the circulation: trypanosomes are regularly lysed by the host immune system as they undergo antigenic variation. Correspondingly, HAT sera contain antibodies which react with trypanosome proteins other than the VSG. Indeed, a preparation of formalin-fixed, ethanol-treated and stained insect-stage *T. brucei* (which lack VSG) was used to create an agglutination test which was reported to have 98% sensitivity and 96% specificity [8]. The ethanol treatment might expose internal proteins. However, this result seems not to have been followed up. An ELISA assay for the related parasite *Trypanosoma evansi*, based on a soluble protein extract, also gave promising results [9] although sensitivity was less than 90% [10]. So far, however, the antigens involved have not been characterized.

Another possibility is to test candidate pure proteins [11]. Two *T. brucei* proteins that were identified by expression library screening, using serum from infected cattle [12], turned out to be cytoskeletal proteins that were also recognised by sera from uninfected mice [13]. A commercially-available Chagas disease ELISA using recombinant protein is sold by Wiener Laboratories (Rosario, Argentina); and another ELISA contains three antigens [14] but in neither case are the identities of the proteins or peptides published. In contrast, in a recent screen of 400 recombinant proteins from the south American trypanosome, *Trypanosoma cruzi*, Cooley et al were able to identify 16 proteins which, in combination, allowed diagnosis of Chagas disease with 100% sensitivity [15]. A multiplex approach was essential since patient responses were heterogeneous.

An alternative to looking for antibodies is to test for the presence of trypanosome proteins. The TrypTECT CIATT consists of latex particles that are coated with a monoclonal antibody to a trypanosome antigen identified only as an “internal protein”. It is assumed that the particles are agglutinated if the same antigen is present in the serum [16]. The TrypTECT CIATT test had only a 3% false-negative rate, and was very specific using controls from trypanosomiasis-free areas serum [16]. In contrast, there was a high apparent false-positive rate from endemic areas; since the positive samples were not tested by PCR the true false-positive rate is unknown [17].

Sera from Leishmaniasis patients were recently used to look for diagnostic antigens in complex extracts, using Western blotting or precipitation followed by mass spectrometry [18], [19]. We have here adopted the same approach of extract screening to look for antigens that might form the basis of a serological test for *T. b. rhodesiense infection*, but have followed up with further tests using recombinant proteins.

## Materials and Methods

### Human Serum Samples

Ethical clearance for this study was obtained from the Ministry of Health and the Uganda National Council for Science and Technology and from the Ethical review board of the University of Heidelberg. Samples were collected from 30 sleeping sickness patients and 30 controls, after written consent, at Namungalwe treatment centre in collaboration with Uganda National HAT control program. The conditions of collection conformed to Good Clinical Practice (GCP) and Good Laboratory Practice (GLP). Immediately after collecting blood, we spotted approximately 200 µl on FTA cards (Whatmann). At the clinic, sleeping sickness infection was confirmed by microscopy. In the lab we processed discs punched from the FTA cards using the FTA purification reagent as instructed by manufacturer. We then tested for T. brucei species and subspecies (*T. b. rhodesiense SRA* gene), using nested PCR and the primers and conditions described in [20]. The controls were healthy volunteers with no clinical signs of trypanosomiasis, malaria or gastrointestinal infection; the absence of trypanosomes in their blood was confirmed by microscopy. Plasma/sera were stored in liquid nitrogen before transfer to the laboratory. Three infection sera were discarded because they had become degraded during transit: SDS-PAGE of these sera revealed no detectable protein.

### Cell Fractionation

All proteomic studies were done using *in vitro* cultivated *Trypanosoma brucei brucei*, strain Lister 427. Procyclic- and bloodstream-form trypanosomes (1×10^9^) were washed in PBS, freeze-thawed to release VSG, then disrupted by grinding with silicon carbide [21]. Organelles were sedimented from the post-nuclear supernatant as described, and the soluble fraction was retained. The glycosomes were then further purified by sucrose gradient centrifugation, as previously described [22], [23].

Cytoskeletons were prepared as described by [24]. 1×10^8^ procyclic form *T. brucei* were harvested by centrifugation at 800 g for 10 min. Washed parasites were resuspended in PIPES buffer (100 mM PIPES (pH 6.9), 2 mM EGTA, 0.1 mM EDTA, 1 mM MgSO_4_, with 1 tablet of ‘complete EDTA –Free’ protease inhibitors cocktail (Roche Applied Science, Mannheim, Germany) per 10 ml). The mixture was centrifuged at 1800×g for 10 minutes, then the supernatant and pellet were analysed by gel electrophoresis.

### Western Blot

One-dimensional SDS-PAGE was performed under denaturing and reducing conditions, transferred onto nitrocellulose membranes (Schleicher & Schüll, Dassel, Germany), which were blocked overnight at 4°C with 5% non-fat milk in Tris Buffered Saline-Tween 20 (TBS-T).

Sera from infected individuals and non-infected controls, diluted 1∶5000 or 1∶1000 in 5% milk (TBS-Tween), were added and incubated for 1 hour at room temperature. After incubation membranes were washed three times (10 min) with TBS-T, and anti-human IgG coupled with Horse Radish Peroxidase (GE Healthcares, UK), diluted 1∶5000 in 5% non-fat milk in TBS-T was added for 1 h. After incubation the membrane was finally washed three times (10 min) and developed according to the ECL detection kit manual (Amersham Biosciences, Freiburg, Germany).

### 2D Electrophoresis

A whole cell extract of procyclic trypanosomes was obtained by pelleting a total of 2×10^8^ cells by centrifugation at 2000 rpm for 10 min. Cells were washed with 1 mL 1x PBS and pellet resuspended in 1 mL 2D sample buffer (7 M Urea, 2 M Thio Urea 4% CHAPS, 1% DTT and ‘complete EDTA –Free’ Protease Inhibitor cocktail (Roche Applied Science, Mannheim, Germany)). The suspension was incubated on ice for 15 min and then centrifuged at 6000 rpm for 5 min to obtain a supernatant. The protein concentration of the extract was determined by the Bradford assay. Sample equivalent to 200 µg protein was loaded on a non-linear immobilized pH gradient IPG strip (3.5–10.0 NL IPG 18 cm; GE Healthcare). The IPG strip was rehydrated at 50 V for 12 h followed by focusing for a total of 50 kVh at 20oC. For protein reduction, the IPG strip was shaken at 100 rpm for 15 min in 0.4 mg/mL DTT, 40 mM Tris-HCl (pH 8.8), 7 M urea, 10% glycerol and 2% (w/v) SDS. Reduced proteins were alkylated by incubating the IPG strip in fresh buffer with the DTT replaced by 5 mg/mL iodoacetamide. The IPG strip was then transferred to the top of a 9–16% polyacrylamide gel (acrylamide/bisacrylamide 37.5∶1, 2.6% cross-linker; gel dimension, 20 cm×20 cm×1 mm) and held in place with 0.5% (w/v) low-melt agarose. Electrophoresis in the second dimension was performed in a Protean II Multicell xi system (Bio-Rad) using 25 mM Tris, 192 mM Glycine, 0.1% SDS buffer at 10°C until the bromophenol blue dye front was 1 cm from the bottom of the gel. Thiol groups were subsequently blocked by incubating with 100 ml of a solution containing Tris-HCl (50 mM) pH 6.8, urea (6 M), glycerol (30% v/v), SDS (2% w/v), iodoacetamide (2.5% w/v) and a trace of Bromophenol Blue for 5 min. Three parallel 2-dimensional gels were made. Two were separately subjected to western blotting by probing with pooled infected and control sera. The other gel was stained with Coomassie brilliant blue and antigen spots excised for analysis by mass spectrometry.

### Gene Cloning and Expression

Proteins were expressed as His-or glutathione-S-transferase (GST) fusions, and purified according to standard protocols. Briefly, the *HSP70* (Tb927.7.1030) and *PFR1* (Tb927.3.4290-4330) open reading frames were amplified by PCR, cloned into pGEMT-easy, then transfer cloned into the pGEX-4T2 expression vector. Expression of protein was induced by addition of IPTG and cells were lysed in PBS-0.4M EDTA, 200 mM PMSF, and ‘complete EDTA–free’ protease inhibitor cocktail (Roche Applied Science, Mannheim, Germany). The proteins were purified on glutathione-Sepharose and eluted with reduced glutathione. The open reading frames of histones H3 and H2B were cloned as *B*gl II- *Hind* III fragments into pQEA. The rhodesian open reading frame [25] was cloned as a *Bam* HI- *Hind*D III fragment into pQE30 or pET28a. We also similarly cloned fragments encoding 100 amino acids of *Tb*NT2 (Tb927.2.6150), *Tb*NT5 (Tb927.2.6240), and *Tb*NT10 (Tb09.160.5480). The fragment - residues 207–309 for TbNT10 and homologous segments of NT2 and NT5 - is predicted to be extracellular by Bioedit and Protein Toolbox programs. Expression of the NT2 and NT5 fragments was too poor for further use. The other His-tagged proteins were purified by Nickel affinity chromatography (Qiagen).

## Results

### Immunoblotting of Sub-Cellular Fractions

**Figure 1:**
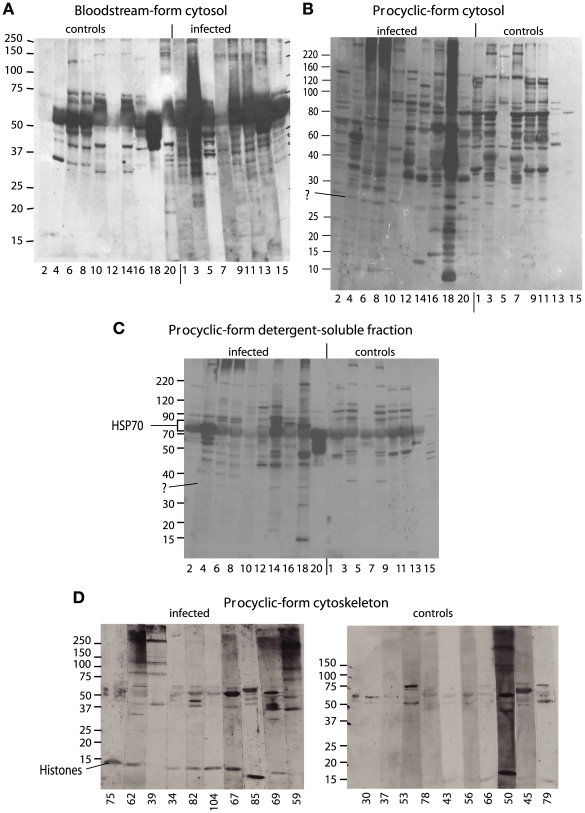
Interaction of human sera with trypanosome fractions. Trypanosome extracts were analysed by Western blotting using sera from sleeping sickness patients (left) or controls (right) diluted at 1∶1000. Bands at the positions indicated were analysed by mass spectrometry; the identified protein is indicated. Serum sample numbers are beneath the lanes and marker positions (kD) are shown to the left. (A) Bloodstream-form cytosol. (B) Procyclic-form cytosol. (C) Procyclic-form detergent-soluble fraction. (D) Procyclic-form cytoskeleton fraction.

**Figure 2:**
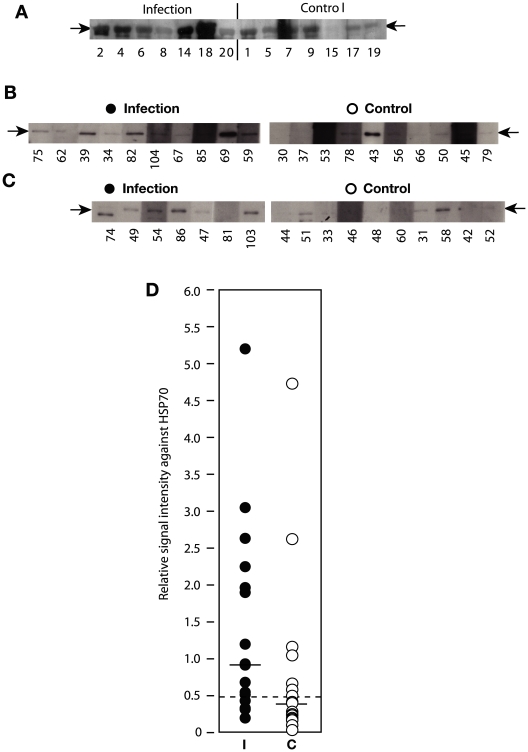
Interaction of human sera with recombinant *T. brucei* HSP70. (A) *T. brucei* HSP70 was produced in *E. coli* as a fusion with glutathione-S-transferase (GST). Panel (A) shows the results using the serum batch 1, and panels (B) and (C) different pairs of Western blots for serum batch 2. (D) Reactivity of all sera against HSP70. The signal of the HSP70 band was measured, and background from immediately above and below the band was subtracted. In each experiment, 20 control sera and 20 infection sera were examined, and the average signal was calculated. The result for each serum was then calculated relative to the average. Each spot represents the average for 3 or 4 Western blots.

Our aim was to find a set of trypanosome proteins that are recognised by sera from patients with African sleeping sickness, but not by control sera from the same region. Sera were obtained from *T. b. rhodesiense* patients who presented at the sleeping sickness clinic in Namungalwe Health centre, Eastern Uganda, and from volunteer healthy controls from the same area (see Methods). Two batches of sera were used: batch 1 (numbered 1–20 in Figures 1A, 1B and 1C and Figure 2A) and, when these were exhausted, batch 2 (numbered above 20, Figure 1D and Figures 2B, 2C).

A variety of different extracts was prepared from cultivated *T. b. brucei*, and analysed by western blotting using the sera. For extract preparation we chose to use a strain of *T. b. brucei* that grows to relatively high densities in culture, even as bloodstream forms, since this is a prerequisite for large-scale cell fractionation and proteomic analysis. The subspecies difference should not affect the sensitivity, since *T. b. rhodesiense* differs from *T. b. brucei* only by the presence of a single protein (SRA) [6].

First, we examined a cytosolic fraction from bloodstream trypanosomes. These blots (Figure 1A) were dominated by a band of about 60 kDa - perhaps a VSG, since VSGs have molecular weights in this region. This band was recognised by control and infection sera. Glycosomes from bloodstream forms, which contain abundant glycolytic enzymes, were also examined. Very few, faint bands were seen but there was one extremely faint band of about 37 kDa that appeared to be recognised only by infection sera (not shown). By aligning the blots with the protein stain, we estimated that this band could either be phosphoglycerate kinase (PGKC), or another protein of similar molecular weight.

Using procyclic forms, to eliminate the strong VSG band, we first examined the cytosolic fraction. The human sera reacted with many bands, but none was preferentially present in the sleeping sickness samples (Figure 1B). We also prepared cytoskeletons by detergent extraction. Using the supernatant (soluble) fraction, two areas at 70 kDa and 35 kDa gave potentially specific reactivity (Figure 1C). Both were examined by mass spectrometry. The 70 kDa band was identified as HSP70; we were unable to identify the 35 kDa protein. The cytoskeleton fraction had a prominent reactivity below 20 kDa (Figure 1D). This contained predominantly histone H2B, although the other core histones (H3 and some H2A and H4) were also detected.

Since the Western blots were yielding predominantly abundant, highly-conserved proteins, we attempted to increase the sensitivity using 2D gels. This time, we probed matched Western blots with pooled infection and control sera. A few spots seemed to be specific for infection sera (Supplementary Figure S1), so proteins in the relevant area were identified by mass spectrometry. The only peptides that could be assigned – apart from HSP70 - came from abundant mitochondrial proteins. These seemed unlikely to be useful as diagnostics since they are evolutionarily conserved and are preferentially expressed by procyclic-form trypanosomes.

In our Western blotting we could only detect immune responses to denatured proteins. In order to extend out analysis to native proteins, we cross-linked antibodies from pooled sera (batch 1) to beads, and immunoprecipitated proteins from procyclic trypanosome extracts. Procyclic extracts were chosen both to avoid the abundant VSG, and because of the previously-reported use of procyclic extract in an agglutination test. We compared the immunoprecipitates by SDS-PAGE, and detected no differences at all. We concluded that if any other proteins were specifically immunoprecipitated by infection sera, the signal was masked by the background from highly abundant proteins.

### Analyses of Recombinant Proteins

Our analyses had yielded four candidate antigens: HSP70, histone H2B, histone H3, and PGKC. To find out whether any of these proteins could be a component of a diagnostic, we tested purified proteins for reactivity with the human sera. Purified PGKC was available, and we expressed HSP70 as a recombinant protein, fused to glutathione S transferase (GST), and also made His-tagged histone H2B and histone H3. In addition, we tested some other available proteins: paraflagellar rod protein 1 (PFR1-GST), and His-tagged *Tb*NT10 and rhodesain, chosen either because they were candidate diagnostics for *T. cruzi*, or because they are potentially exposed on the trypanosome surface. The results suggested that HSP70, but not the other proteins, was preferentially recognised by the infection sera. (Note that PFR1-GST was not recognised, ruling out a reaction of the sera with GST.) Figure 2A shows Western blot results with batch 1 sera, and Figures 2B and 2C those with batch 2. The complete blots are shown in Supplementary Figure S2. Although the protein preparations appeared pure by Coomassie staining, many of the sera showed background reactivity with contaminating *E. coli* proteins. This resulted in high backgrounds in ELISA assays (not shown). Some sera also consistently gave high background on the membrane. To measure specific reactivity to *Tb*HSP70, we therefore focussed only on the relevant Western blot band. To quantitate the results, the batch 2 sera were tested three times, each time in batches of 10 controls and 10 infected; to normalise the results for different blots, the 70 kDa signal strengths were calculated relative to the average signal for all 20 sera in the experiment. The results are plotted in Figure 2D and details for each serum in Supplementary Figure S3. The median signal in the infection sera was 2.4 times higher than that from control sera (horizontal lines), but due to the large range in reactivity of the sera, the difference was not statistically significant (Wilcoxon ranking test). Diagnostic tests usually involve selecting a cut-off of reactivity: samples above the cut-off are regarded as positive, and those below it, negative. Using a cut-off of 0.5 relative signal intensity (dotted line in Figure 2D), the Western blot would have diagnosed 70% of the sleeping sickness patients, with a false-positive result for 35% of controls.

## Discussion

Our proteomic screen was set up to identify antigenic proteins present in HAT patient sera, with a focus on *T. b. rhodesiense*. Our approach enabled us, in effect, to screen many hundreds of proteins present in various *T. b. brucei* subcellular fractions. Our most important conclusion was that – with the possible exception of *Tb*HSP70 – none of the *T. brucei* proteins that we detected by Coomassie staining was likely to be useful as a diagnostic antigen for sleeping sickness. (For the 1D-electrophoresis studies, bearing in mind that some purification was done, a Coomassie detection limit of about 20 ng [26], [27] translates into 0.01% to 0.2% of total cell protein.) This rules out a large proportion of the recombinant proteins that have been produced so far, since there has been a strong focus on abundant metabolic enzymes.

From our initial screening, HSP70, histone H2B, histone H3, and PGKC were selected for further analysis of cloned and purified products. In addition we cloned proteins not identified during the screening, but considered to be potentially antigenic. TbNTs play a central role in purine salvage due to the inherent inability of trypanosomes to effect *de novo* biosynthesis. The first step in this salvage entails import of purines across the cell membrane by nucleoside or nucleobase transporters [28]. Due to their crucial role, we speculated that many of the TbNTs must be expressed by bloodstream-form *T. brucei*. Rhodesain was included in the analysis because its counterpart in *T. congolense* was shown to be immunogenic in cattle [29].

No specific reactivity was found for histones H2B and H3, PGKC, PFR1, rhodesain and TbNT10, but we found that most sera from sleeping sickness patients reacted with *T. brucei* HSP70. Some control sera also reacted: this could be a cross reaction, or might indicate previous exposure to non infective trypanosomes, such as *T. b. brucei*, circulating alongside *T. b. rhodesiense* within the tsetse fly and the domestic animal reservoir. Misdiagnosis or the controls, due to the low sensitivity of the available parasitological tests, is unlikely since all controls remained non-parasitaemic, with no signs suggestive of HAT, during follow-up checks. Since our samples came from only 60 individuals (30 patients and 30 controls), testing with a larger panel of sera will be required to determine the sensitivity and selectivity of the interaction with HSP70. In addition, our study did not investigate whether the presence of other infections (e.g. malaria, Leishmaniasis) would also result in a positive signal.

Based on the results shown here, we conclude that although *Tb*HSP70 cannot be used alone to diagnose trypanosomiasis, it might be a useful component of a multiplex diagnostic containing several immunogenic proteins. Results with *T. cruzi* and diagnostics have similarly suggested that several antigens are required. The antigen panel chosen by Cooley et al. [15] was biased by design towards abundant proteins, and the final list included mitochondrial HSP70, a ribosomal protein, a paraflagellar rod protein and two other flagellar proteins, as well as glycosomal phosphoenol pyruvate carboxykinase. A different *T. cruzi* study detected antibodies to HSP70 in chronically-infected Chagas patients [30]. *Leishmania* proteomics studies also identified abundant proteins: HSP70, another chaperone (Grp78), a surface protease and translation factors [18], [19]; one of the studies also found several glycosomal proteins. The potential of these *Leishmania* proteins as diagnostics was however not confirmed using pure protein.

The approach undertaken in this study had clear limitations. Bands or spots were chosen by Western blotting, then proteins with equivalent migration were identified from another gel. If a signal is seen on a blot, it could arise from any protein migrating at that position, but mass spectrometry detects only the most abundant ones, which may not react with the sera at all. Similarly, our immunoprecipitation experiments were limited to procyclic-form extracts. We therefore suggest that more thorough purification of different fractions, taken from both bloodstream and procyclic forms, and followed by quantitative mass spectrometry, will be required to identify good diagnostic antigens for *T. b. rhodesiense* infection.

## Supporting Information

Figure S1 2-dimensional gel electrophoresis and Western blotting. Extracts were separated by isoelectric focussing (pH shown above the gel and blots) then by size (size markers on the right). Duplicate Western blots were probed with pooled infection (A) or control (B) sera (sera 1–20) and compared. Areas containing proteins that reacted specifically with infection sera were excised from a Coomassie-stained gel (C) and identified by mass spectrometry. Proteins identified were: 1, 4, 5 - HSP70 2 - Tb927.8.6170 - transketolase 6 - Tb927.6.2790 - threonine dehydrogenase 7 - Tb11.01.5860 - HSP60 family The proteins identified for 2, 6 and 7 are specific to procyclics so are not good diagnostic candidates. The patchy background on the blots was see in two independent experiments, so is presumably a characteristic of the sera.
(3.47 MB EPS)

Figure S2 Reactions of sera with purified *T. brucei* HSP70: whole Western blots. *T. brucei* HSP70 was produced in *E. coli* as a fusion with glutathione-S-transferase (GST). Purified protein (H) is shown next to the marker on a Coomassie-stained gel, and Western blot strips stained using infection sera diluted to 1∶1000. (A) Blot shown in Figure 2A. (B) Upper blots shown in Figure 2B, run and detected together. (C) Lower blots shown in Figure 2B, run and detected together. A typical Coomassie stain of the purified protein is shown to the left of Figure 2B. Note the cross-reaction with *E. coli* proteins which cannot be seen on the Coomassie stain. This background precluded the use of ELISA to quantitate reactivity with HSP70. Also, some sera consistently gave dark backgrounds on the membranes. Sera 32, 55 and 107 were checked by SDS-PAGE and found to contain no protein, so results with these were discarded.
(2.88 MB EPS)

Figure S3 Details of the reactivity of human sera (batch 2) with *T. brucei* HSP70. Results are arithmetic mean and standard deviation for 3 or 4 experiments.
(0.44 MB EPS)
